# A Retrospective Study Comparing the Operative Outcomes of Extraperitoneal, Retrorectus Access Laparoscopic and Robotic‐Assisted Ventral Hernia Repairs

**DOI:** 10.1002/wjs.12580

**Published:** 2025-04-09

**Authors:** Vivek Bindal, Dhananjay Pandey, Shailesh Gupta, Priyanka Agarwal, Usha Dudeja Bindal

**Affiliations:** ^1^ Bariatric & Robotic Surgery Max Institute of Minimal Access Max Super Speciality Hospital Vaishali Ghaziabad India; ^2^ Department of Biochemistry Post Graduate Institute of Child Health Noida India

**Keywords:** eTEP, eTEP‐RS, pain, quality of life, robotic‐assisted surgery, ventral hernia repairs

## Abstract

**Background:**

This retrospective study aims to evaluate the operative outcomes of laparoscopic and robotic extraperitoneal repair of abdominal wall defects via enhanced view total extraperitoneal (eTEP) retrorectus space access.

**Methods:**

A medical chart review was performed on consecutive eTEP cases from our unit, focused on collecting perioperative outcomes.

**Results:**

One hundred and twenty cases were collected, 73 in the robotic group and 47 in the laparoscopic group. Approximately 38% of the robotic and 64% of the laparoscopic arms required component separation. In the overall population (irrespective of defect size and technique used), the robotic arm versus the laparoscopic arm had (a) significantly higher (*P* < 0.001) mean hernia defect, (b) shorter operating time (*P* < 0.001), (c) significantly fewer postoperative complications (*P* = 0.039), (d) significantly fewer pain scores at 24 hours and 14 days postsurgery (*P* = 0.002 and *P* < 0.001, respectively), and (e) better patient well‐being scores (*P* = 0.001). The length of hospital stay and analgesic usage were comparable. A subgroup analysis by defect size (< 7 cm, 7–10 cm, and > 10 cm) revealed that approximately 51% of patients needed component separation in the laparoscopic group for defects < 7 cm, and 100% of patients needed it for hernias > 7 cm in this group. In the robotic group, no patient (0%) needed component separation for defects < 7 cm, and approximately 43% needed it for defects > 7 cm.

**Conclusions:**

This study reports encouraging short‐term outcomes for the robotic‐assisted eTEP approach in Indian settings. The robotic‐assisted approach has the potential to reduce the requirement of component separation in patients with large ventral hernia defects. However, future prospective, randomized studies with long‐term follow‐up on recurrence will be needed to validate our findings.

## Graphical abstract

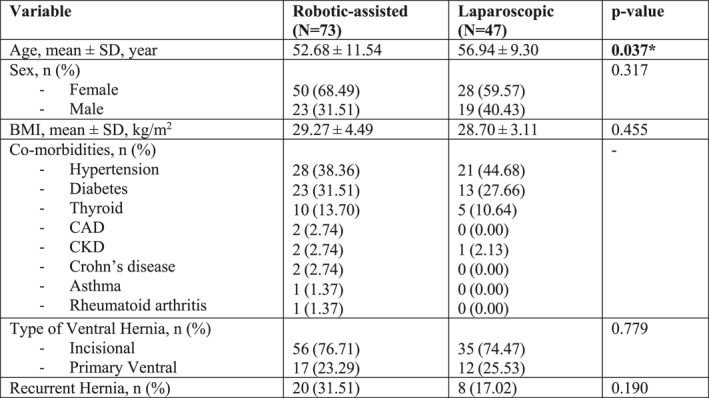

## 1 Introduction

Ventral hernias, whether primary or incisional, represent serious public health concerns due to their high treatment costs, prevalence, and variability in surgical management [1]. Ventral hernia repair (VHR) is one of the most common procedures performed by general surgeons, accounting for around 400,000 surgeries and a $3 billion economic burden in the United States annually [2]. Although there are several options for treating hernias, ranging from open surgeries to alternative minimally invasive procedures, the best approach is yet to be determined [3]. The widespread adoption of minimally invasive procedures has led to a surge in new techniques, such as the eTEP approach [4, 5, 6]. This approach was first designed for laparoscopic inguinal hernia repair in 2012 but was later modified for VHR [5, 6]. The eTEP‐VHR offers several advantages such as sublay mesh placement in the retromuscular space without the need for traumatic fixation and the ability to deal with a very large hernia by executing a posterior component separation with TAR [2]. Additionally, it prevents the mesh from coming into contact with the abdominal viscera and offers a plane to reinforce the abdominal wall [3, 7]. Furthermore, in a study, none of the patients reported chronic pain or dissatisfaction with the eTEP procedure. In contrast, the use of multiple sutures or tacks in other procedures may induce pain during the immediate postoperative period as well as chronic pain owing to nerve entrapments [3, 8]. A study found that the eTEP approach allows for greater flexibility in repairing a broad range of abdominal wall defects [9].

However, the eTEP approach is technically very challenging; creating the retrorectus space, component separation, and suturing all require advanced skill sets. Furthermore, the laparoscopic extraperitoneal approach poses major ergonomic challenges for the operating surgeon and restricts the degrees of freedom [9]. Therefore, the effective implementation of the eTEP approach for VHR requires advanced technical skills in minimally invasive surgery [9]. Additionally, the delicate intricacy of this technique makes it challenging for other surgeons to replicate [6]. Robotic‐assisted surgical techniques, on the other hand, offer greater degrees of freedom, improve ergonomics, and allow for finer motions that are difficult to accomplish with traditional methods [10, 11, 12]. Robotic technology has the potential to improve the reproducibility of VHR via the eTEP approach, benefiting both patients and surgeons in the long run. Moore et al. found that by utilizing the robotic platform, inexperienced operators were able to accomplish surgical tasks more quickly and precisely, demonstrating the transferability of surgical skills to increasingly difficult tasks [13]. This learning advantage persisted even when the operator had to perform a task under stressful conditions [9, 13].

There is limited clinical information to compare laparoscopic and robotic‐assisted eTEP and TAR procedures in VHR. Therefore, we undertook this retrospective study to compare the operative and postoperative outcomes of laparoscopic and robotic‐assisted extraperitoneal repairs of abdominal wall defects.

## 2 Material and Methods

This study was carried out at the Max Institute of Minimal Access, Bariatric, and Robotic Surgery, India. A retrospective chart review was carried out for patients with ventral or incisional ventral hernia who underwent VHR using a laparoscopic or robotic‐assisted extraperitoneal approach between June 2021 and October 2023. During this period, 120 patients underwent extraperitoneal repair (eTEP with or without TAR). All of these patients were included in the analysis. As per our institutional practice, we use eTEP (laparoscopic or robotic) for complex ventral hernias (defects larger than 5 cm or incisional hernias). Usually, the tension on the posterior rectus sheath (PRS)–peritoneum complex during closure determines whether or not to perform a TAR. Our preoperative workup includes a detailed history and physical examination, as well as standard laboratory tests. A CT scan of the abdomen and pelvis is ordered for preoperative planning.

Baseline variables including age, sex, body mass index (BMI), comorbidities, type of hernia repair (incisional or ventral hernia), and information on recurrent hernias were taken from the medical records to collect data. Intraoperative and postoperative variables, such as the size of the hernia defect (length, width); the laparoscopic or robotic‐assisted surgical approach used, such as eTEP Rives–Stoppa (eTEP‐RS), eTEP‐RS unilateral transversus abdominis release (eTEP‐RS U/L TAR), and eTEP‐RS bilateral TAR (eTEP‐RS B/L TAR); total operating time; mesh size; length of hospital stay; analgesics usage; postoperative complications; pain scores at 6 h, 24 h, and 14 days after surgery patient well‐being scores within 14 days of surgery; and number of readmissions were collected from surgery notes. Pain was assessed as per routine clinical practice using a numerical rating scale (NRS) ranging from 0 to 10, with "0″ reflecting no pain and "10″ indicating the worst pain imaginable [14]. For assessing patients' well‐being and perception of their health, a visual analog score (EQ‐VAS) from the EuroQol 5‐dimensional instrument with 3‐level classification system (EQ‐5D‐3L) was used. This score runs from 0 to 100, with 0 indicating the worst health and 100 indicating the best health, as reported and perceived by the patient.

The da Vinci Xi surgical system (Intuitive Surgical, Sunnyvale, CA, USA) was utilized for robotic‐assisted surgery. It consists of a 3D vision system and EndoWrist instruments with 7 degrees of freedom that simulate dexterity and a range of movement, resulting in great precision and flexibility. All surgeries were conducted by a single skilled surgeon.

The statistical analysis of the quantitative variables was summarized as the arithmetic mean with standard deviation (SD). Frequencies and percentages were used to summarize categorical data. Pearson chi‐square test or Fisher's exact test, as appropriate, was used to compare frequencies between the groups. Students' t‐test was used to compare differences in means between the robotic‐assisted and laparoscopic groups. A two‐sided *P* < 0.05 was considered statistically significant. Statistical analysis was performed using Stata 16.0 statistical software (StataCorp LLC, Texas, USA).

### 2.1 Surgical Technique

Initial entry using an optical trocar into the left retrorectus space was made in the upper abdomen approximately 5–6 cm lateral from the midline, and the retrorectus space was expanded further using scope dissection.

### 2.2 Robotic Arm

Two 8‐mm secondary robotic trocars are placed just medial to the linea semilunaris at the level of the umbilicus and left lumbar region, avoiding any injury to NV bundles. The robotic patient cart comes from the right side of the patient and is docked. Crossover is performed onto the opposite retrorectus space in the upper abdomen. Bilateral retrorectus dissection is performed further caudally. In this process, the hernia sac is encountered and dealt with. Every effort is made to preserve as much sac as possible.

This process of division of PRS in the midline continues until the arcuate line, where PRS becomes continuous with the fascia transversalis to enter the space of Retzius and Bogros. Posteriorly, a tension‐free closure of the PRS–peritoneal complex is performed with absorbable barbed suture after lowering the pressure to 6–10 mm of Hg. The preserved sac is utilized to aid in posterior closure, possibly preventing transversus abdominis release (TAR) in some cases. Anterior defect and linea alba closure are performed using nonabsorbable barbed suture. In cases where closure of the PRS–peritoneal complex cannot be achieved without tension, TAR is required.

Dimensions of the potential space created are measured, and a macroporous polypropylene mesh is placed. No mesh fixation is performed routinely, and a drain is placed selectively.

### 2.3 Laparoscopic arm

After creating the retrorectus space on the ipsilateral side by blunt dissection, one 10‐mm and one 5‐mm secondary trocars are placed under needle guidance at the level just medial to the linea semilunaris.

After crossover, one 5‐mm trocar is placed in the right retrorectus space under vision. Dissection in the retrorectus space, as well as closure of defects, is continued in a similar fashion as described in the robotic technique. During closure of the anterior rectus sheath, sometimes one extra 5‐mm port is placed at the level of the umbilicus in the right retrorectus space to aid in closure of the anterior rectus sheath cranially.

Thus, in the laparoscopic arm, 4–5 ports were used, whereas in the robotic arm, three ports were used.

## 3 Results

### 3.1 Baseline Characteristics

A total of 120 consecutive cases who underwent retrorectus access ventral hernia repairs were identified, including 73 in the robotic group and 47 in the laparoscopic group. The mean age was lower in the robotic group. The majority of hernia defects in both groups were incisional, whereas approximately 31.51% of defects in the robotic arm and 17.02% in the laparoscopic arm were recurrent. Table 1 summarizes the research population’s baseline characteristics.

**TABLE 1:** Baseline characteristics of the study population.

Variable	Robotic (*N* = 73)	Laparoscopic (*N* = 47)	*p*‐value
Age, mean ± SD, year	52.68 ± 11.54	56.94 ± 9.30	**0.037** **^a^**
Sex, *n* (%)	50 (68.49)	28 (59.57)	0.317
Female	23 (31.51)	19 (40.43)	—
Male	—	—	—
BMI, mean ± SD, kg/m^2^	29.27 ± 4.49	28.70 ± 3.11	0.455
Comorbidities, *n* (%)	28 (38.36)	21 (44.68)	—
Hypertension	23 (31.51)	13 (27.66)	
Diabetes	10 (13.70)	5 (10.64)	
Thyroid	2 (2.74)	0 (0.00)	
CAD	2 (2.74)	1 (2.13)	
CKD	2 (2.74)	0 (0.00)	
Crohn's disease	1 (1.37)	0 (0.00)	
Asthma	1 (1.37)	0 (0.00)	
Rheumatoid arthritis	—	—	
Type of ventral hernia, *n* (%)	56 (76.71)	35 (74.47)	0.779
Incisional	17 (23.29)	12 (25.53)	
Primary ventral	—	—	
Recurrent hernia, *n* (%)	20 (31.51)	8 (17.02)	0.190

*Note:* *p* < 0.05 was considered significant. *p* < 0.01 was considered highly significant.

Abbreviations: BMI: body mass index; CAD: coronary artery disease; CKD: chronic kidney disease; SD: standard deviation.

^a^ Significant value.

### 3.2 Operative Outcomes

#### 3.2.1 Outcomes of the Overall Population

The most frequently employed approach for the robotic group was eTEP‐RS (61.64%), followed by eTEP‐RS U/L TAR (34.24%) and eTEP‐RS B/L TAR (4.10%), respectively. The most commonly used technique in the laparoscopic group was eTEP‐RS U/L TAR (44.68%), followed by eTEP‐RS (36.17%) and eTEP‐RS B/L TAR (19.14%), respectively. The operative outcomes of the study population are summarized in Table 2. The robotic group had a significantly higher (*P* < 0.001) mean length, width, and area of hernia defect than the laparoscopic group. The operating time was significantly longer in the laparoscopic group compared to the robotic group (*P* < 0.001). The length of hospital stay, as well as the number and duration of analgesic usage, were comparable across the robotic and laparoscopic groups. Pain scores at 24 h (*P* = 0.002) and within 14 days (*P* < 0.001) of surgery were significantly better in the robotic group. Similarly, the patient well‐being score was significantly higher in the robotic group (*P* = 0.001). The laparoscopic group reported significantly higher postoperative complications. One patient in the laparoscopic group required readmission.

**TABLE 2:** Operative outcomes of the overall population.

Variable	Robotic (*N* = 73)	Laparoscopic (*N* = 47)	*p*‐value
Technique‐ eTEP‐RS, *n* (%)	45 (61.64)	17 (36.17)	**0.006** **^a^**
‐ eTEP‐RS U/L TAR, *n* (%)	25 (34.24)	21 (44.68)	0.251
‐ eTEP‐RS B/L TAR, *n* (%)	3 (4.10)	9 (19.14)	**0.009** **^a^**
Total operating time, mean ± SD, min	152.30 ± 52.22	194.94 ± 54.73	**<0.001** **^a^**
Length of hernia, mean ± SD, cm	11.04 ± 4.25	7.21 ± 3.24	**<0.001** **^a^**
Width of hernia, mean ± SD, cm	8.07 ± 3.17	5.21 ± 1.61	**<0.001** **^a^**
Area of hernial defect, mean ± SD, cm^2^	79.49 ± 60.36	32.85 ± 20.82	**<0.001** **^a^**
Length of mesh, mean ± SD, cm	28.63 ± 2.05	27.96 ± 1.92	0.077
Width of mesh, mean ± SD, cm	20.68 ± 5.27	23.13 ± 6.58	**0.028** **^a^**
Length of hospital stay, mean ± SD, days	2.53 ± 0.76	2.21 ± 0.74	**0.025** **^a^**
Post‐op complications, *n* (%)	2 (2.74)	6 (12.77)	**0.039** **^a^**
‐ Bruising at port site	0 (0.00)	2 (4.26)	
‐ Intestinal obstruction	0 (0.00)	1 (2.13)	
‐ Retention of urine	0 (0.00)	1 (2.13)	
‐ Seroma	1 (1.37)	2 (4.26)	
‐ AKI, chest infection	1 (1.37)	0 (0.00)	
Clavien–Dindo classification	1 (1.37)	6 (12.77)	**0.014** **^a^**
‐ Grade I	1 (1.37)	0 (0.00)	0.608
‐ Grade II	—	—	—
Number of analgesics used per day prior to discharge, mean ± SD	5.04 ± 1.07	4.89 ± 1.48	0.530
Pain score at 6 h, mean ± SD	5.59 ± 0.66	5.72 ± 0.74	0.304
Pain score at 24 h, mean ± SD	3.90 ± 0.76	4.34 ± 0.69	**0.002** **^a^**
Pain score within 14 days of surgery, mean ± SD	2.11 ± 0.59	2.66 ± 0.56	**<0.001** **^a^**
QoL/patient well‐being score within 14 days of surgery, mean ± SD	85.55 ± 5.62	82.38 ± 4.28	**0.001** **^a^**
Readmission within 30 days after surgery, *n* (%)	0 (0.00)	1 (2.13)	0.392

*Note:* *p* < 0.05 was considered significant. *p* < 0.01 was considered highly significant.

Abbreviations: AKI: acute kidney injury; eTEP‐RS: enhanced‐view totally extraperitoneal Rives–Stoppa; eTEP‐RS B/L TAR: eTEP‐RS bilateral transversus abdominis release; eTEP‐RS U/L TAR: eTEP‐RS unilateral transversus abdominis release; QoL: quality of life; SD: standard deviation.

^a^ Significant value.

#### 3.2.2 Outcomes Based on Defect Size

We also compared the operative outcomes of the study population based on the width of the hernia defect (Table 3). The width was chosen as it is the primary determinant of the need for a component separation technique. In hernias < 7 cm, all patients in the robotic group underwent eTEP‐RS. In the laparoscopic group, approximately 48% underwent eTEP‐RS, and the rest needed component separation. The robotic group had significantly lower total operating time (*P* < 0.001), pain score at 24 h (*P* = 0.006), and pain score within 14 days after surgery (*P* < 0.001) for hernias smaller than 7 cm. Similarly, the QoL/patient well‐being scores 14 days after surgery were significantly higher in the robotic group (*P* = 0.012). For defects of 7–10 cm, 43.5% in the robotic group required component separation, whereas 100% of patients in the laparoscopic group needed component separation. The robotic group had a significantly shorter total operating time (*P* < 0.001) and a lower pain score within 14 days of surgery (*P* = 0.003) than the laparoscopic group. The defect size of more than 10 cm was only repaired via robotic surgery rather than a laparoscopic approach. For the defect size of > 10 cm, 88.89% of patients in the robotic group underwent eTEP‐RS U/L TAR.

**TABLE 3:** Operative outcomes of the study population based on hernia defect size.

Hernia < 7 cm (width)	Robotic (*N* = 32)	Laparoscopic (*N* = 35)	*p*‐value
Total operating time, mean ± SD, min	121.03 ± 34.16	182.60 ± 55.01	**< 0.001** **^a^**
Type of surgery	32 (100.00)	17 (48.57)	—
‐ eTEP‐RS	0 (0.00)	15 (42.86)	
‐ eTEP‐RS U/L TAR	0 (0.00)	3 (8.57)	
‐ eTEP‐RS B/L TAR	—	—	
Length of mesh, mean ± SD, cm	27.91 ± 1.72	27.51 ± 1.79	0.373
Width of mesh, mean ± SD, cm	17.09 ± 1.16	21.03 ± 5.37	**< 0.001** **^a^**
Length of hospital stay, mean ± SD, days	2.38 ± 0.60	2.17 ± 0.74	0.228
Number of analgesics used per day before discharge, mean ± SD	4.88 ± 0.89	4.80 ± 1.62	0.820
Length of analgesic usage, mean ± SD, days	4.91 ± 1.35	5.06 ± 0.89	0.595
Pain score at 6 h, mean ± SD	5.44 ± 0.66	5.66 ± 0.75	0.217
Pain score at 24 h, mean ± SD	3.75 ± 0.71	4.26 ± 0.73	**0.006** **^a^**
Pain score within 14 days of surgery, mean ± SD	2.03 ± 0.53	2.57 ± 0.55	**< 0.001** **^a^**
QoL/patient well‐being score within 14 days of surgery, mean ± SD	85.91 ± 5.52	82.66 ± 4.55	**0.012** **^a^**

**Hernia 7‐10 cm (width)**	**Robotic (N = 23)**	**Laparoscopic (N = 12)**	*p* **‐value**
Total operating time, mean ± SD, min	153.78 ± 55.86	230.92 ± 34.13	**< 0.001** **^a^**
Type of surgery	13 (56.52)	0 (0.00)	—
‐ eTEP‐RS	9 (39.13)	6 (50.00)	
‐ eTEP‐RS U/L TAR	1 (4.35)	6 (50.00)	
‐ eTEP‐RS B/L TAR	—	—	
Length of mesh, mean ± SD, cm	28.61 ± 2.35	29.25 ± 1.69	0.422
Width of mesh, mean ± SD, cm	20.70 ± 4.94	29.25 ± 5.92	**< 0.001** **^a^**
Length of hospital stay, mean ± SD, days	2.70 ± 1.04	2.33 ± 0.75	0.305
Number of analgesics used per day before discharge, mean ± SD	5.13 ± 1.33	5.17 ± 0.90	0.926
Length of analgesic usage, mean ± SD, days	5.35 ± 2.18	5.08 ± 0.28	0.687
Pain score at 6 h, mean ± SD	5.74 ± 0.74	5.92 ± 0.64	0.496
Pain score at 24 h, mean ± SD	4.13 ± 0.80	4.58 ± 0.49	0.090
Pain score within 14 days of surgery, mean ± SD	2.17 ± 0.70	2.92 ± 0.49	**0.003** **^a^**
QoL/patient well‐being score within 14 days of surgery, mean ± SD	85.35 ± 6.27	81.58 ± 3.23	0.067

**Hernia > 10 cm** (width)	**Robotic (N = 18)**	**Laparoscopic (N = 0)**	*p* **‐value**
Total operating time, mean ± SD, min	206.00 ± 19.29	0.00	—
Type of surgery	0 (0.00)	0 (0.00)	—
‐ eTEP‐RS	16 (88.89)	0 (0.00)	
‐ eTEP‐RS U/L TAR	2 (11.11)	0 (0.00)	
‐ eTEP‐RS B/L TAR	—	—	
Length of mesh, mean ± SD, cm	29.94 ± 1.43	0.00	—
Width of mesh, mean ± SD, cm	27.06 ± 3.94	0.00	—
Length of hospital stay, mean ± SD, days	2.61 ± 0.49	0.00	—
Number of analgesics used per day before discharge, mean ± SD	5.22 ± 0.92	0.00	—
Length of analgesic usage, mean ± SD, days	5.89 ± 1.66	0.00	—
Pain score at 6 h, mean ± SD	5.67 ± 0.47	0.00	—
Pain score at 24 h, mean ± SD	3.89 ± 0.74	0.00	—
Pain score within 14 days of surgery, mean ± SD	2.17 ± 0.50	0.00	—
QoL score/patient well‐being score within 14 days of surgery, mean ± SD	85.17 ± 4.82	0.00	—

*Note:* *p* < 0.05 was considered significant. *p* < 0.01 was considered highly significant.

Abbreviations: eTEP‐RS: enhanced‐view totally extraperitoneal Rives–Stoppa; eTEP‐RS B/L TAR: eTEP‐RS bilateral transversus abdominis release; eTEP‐RS U/L TAR: eTEP‐RS unilateral transversus abdominis release; QoL: quality of life; SD: standard deviation.

^a^ Significant value.

When the operative outcomes of the robotic group (> 10 cm) were compared to those of the laparoscopic group (hernia width 7–10 cm), it was discovered that the total operating time (*P* = 0.020), pain score at 24 h (*P* = 0.009), and pain score within 14 days after surgery (*P* < 0.001) were significantly lower in the robotic group (Table 4). Similarly, the QoL scores within 14 days after surgery were significantly higher in the robotic group (*P* = 0.037).

**TABLE 4:** Comparison of operative outcomes of robotic (hernia >10 cm) and laparoscopic (hernia 7–10 cm) eTEP with TAR.

Variable	Robotic hernia >10 cm (*N* = 18)	Laparoscopic hernia 7–10 cm (*N* = 12)	*p*‐value
Total operating time, mean ± SD, min	206.00 ± 19.29	230.92 ± 34.13	**0.020** **^a^**
Type of surgery	0 (0.00)	0 (0.00)	—
‐ eTEP‐RS	16 (88.89)	6 (50.00)	
‐ eTEP‐RS U/L TAR	2 (11.11)	6 (50.00)	
‐ eTEP‐RS B/L TAR	—	—	
Length of mesh, mean ± SD, cm	29.94 ± 1.43	29.25 ± 1.69	0.252
Width of mesh, mean ± SD, cm	27.06 ± 3.94	29.25 ± 5.92	0.248
Length of hospital stay, mean ± SD, days	2.61 ± 0.49	2.33 ± 0.75	0.243
Number of analgesics used per day before discharge, mean ± SD	5.22 ± 0.92	5.17 ± 0.90	0.875
Length of analgesic usage, mean ± SD, days	5.89 ± 1.66	5.08 ± 0.28	0.119
Pain score at 6 h, mean ± SD	5.67 ± 0.47	5.92 ± 0.64	0.245
Pain score at 24 h, mean ± SD	3.89 ± 0.74	4.58 ± 0.49	**0.009** **^a^**
Pain score within 14 days of surgery, mean ± SD	2.17 ± 0.50	2.92 ± 0.49	**<0.001** **^a^**
QoL/patient well‐being score within 14 days of surgery, mean ± SD	85.17 ± 4.82	81.58 ± 3.23	**0.037** **^a^**

*Note:* *p* < 0.05 was considered significant. *p* < 0.01 was considered highly significant.

Abbreviations: eTEP‐RS: enhanced‐view totally extraperitoneal Rives–Stoppa; eTEP‐RS B/L TAR: eTEP‐RS bilateral transversus abdominis release; eTEP‐RS U/L TAR: eTEP‐RS unilateral transversus abdominis release; QoL: quality of life; SD: standard deviation.

^a^ Significant value.

## 4 Discussion

The technique presented in this study was first described by Belyansky et al. [6, 9]. This group described the first series of laparoscopic and robotic‐assisted eTEP in 2017 and 2018, respectively, demonstrating the feasibility and safety of this retrorectus access VHR approach [6, 9]. A key benefit of eTEP‐RS, especially in cases of large incisional hernias, is that TAR, a posterior component separation method, can be performed if defect closure is not possible because the dissection space remains unaltered [3, 7, 15]. Additionally, the entire weakened area of the abdominal wall along the incision can be reconstructed and covered by a mesh. The use of robotic surgery has improved the ability to operate on complex patients by optimizing certain maneuvers, including saving more peritoneum on the posterior layer and the ability to better suture close larger anterior defects [16]. Given these considerations, our study provided insight into the operative outcomes of robotic‐assisted and laparoscopic eTEP‐RS and eTEP‐TAR procedures in routine clinical practice. To the best of our knowledge, this is the first study from India to report on the surgical outcomes of robotic‐assisted and laparoscopic eTEP‐RS and eTEP‐TAR procedures.

Our study population's demographic characteristics, such as mean age, BMI, sex distribution, and ventral hernia type, were consistent with the findings of previous studies [3, 17, 18]. The mean length, width, and area of the hernia defect were higher in the robotic group in our study. This reveals our practice of gravitating toward using robotic platforms for more complex ventral hernias. A previous study found that the robotic‐assisted eTEP arm had larger hernia defects (7.1 vs. 5.5 cm, *P* < 0.001) than laparoscopic eTEP [16].

We have divided the discussion of outcomes into two parts: (a) outcomes of the overall population and (b) outcomes based on defect size.

- Outcomes of the overall population: The total operating time was lower in the robotic arm compared to the laparoscopic arm of our study. A study by Lu et al. was one of the first studies to compare robotic eTEP with laparoscopic eTEP [16]. In contrast to our study findings, this study reported significantly higher operative time in the robotic group compared to the laparoscopic group. Lu et al. hypothesized that the longer operative time in their study was probably due to larger defect size and more complex patients in the robotic arm. In addition, they also included the experience of their robotic learning curve period [16]. In our case, the defect size was greater than the sizes reported by Lu et al. for both the robotic and laparoscopic groups. Essentially, the mean defect size in our robotic group was greater than in their study, and similarly, the defect size in our laparoscopic group was greater than in their study. This also probably reflects our relatively longer experience with robotic programs (using them since 2011) as well as the high volumes of robotic cases. The lower operating time may also be attributed to the lower requirement of component separation in the robotic group versus the laparoscopic group (38% vs. 64%). There was a significant difference (*P* = 0.039) in postoperative complications between the robotic and laparoscopic groups in our study. These findings are consistent with those from a previous study, which found that the laparoscopic‐assisted eTEP‐RS approach had significantly higher complications during or after 30 days after surgery than the robotic approach [16]. A meta‐analysis, however, demonstrated no difference in short‐term postoperative complications between the robotic and laparoscopic‐assisted eTEP‐RS approaches (OR = 0.94, 95% CI 0.49–1.81, *P* = 0.85) [19]. In our study, the pain score at 24 h and within 14 days after surgery was significantly lower in the robotic group. Similarly, in a previously published report, 56% of patients in the laparoscopic arm had clinically significant pain 4 weeks after VHR [6, 20]. We believe the findings of more postoperative complications and worse pain scores in the laparoscopic arms may again be attributed to greater requirements of component separation. The length of hospital stay between the laparoscopic and robotic technique arms in our study was comparable. This is consistent with the reported literature [16]. A 2024 meta‐analysis by Tran et al. reported the most comprehensive comparison of outcomes among open, laparoscopic, and robotic ventral hernia repairs [21]. This meta‐analysis did not report a significant difference between robotic and laparoscopic hernia repairs in terms of length of hospital stay, intraoperative complications, surgical site infections, and surgical site recurrence at 30 days, whereas operative time was longer in the robotic group. They did not report their outcomes on pain and quality of life. The 1‐year recurrence rate was comparable between the robotic and laparoscopic arms [21]. However, this meta‐analysis did not specify the size of the hernia defect and the type of technique used, whereas our study's hypothesis primarily rests on defect size and technique.
- Outcomes based on defect size

When we compared operative outcomes according to hernia size, larger hernia defects in the robotic subgroup were repaired with eTEP‐RS, without the need for component separation (TAR), compared to the laparoscopic subgroup. No patient in the robotic subgroup with a defect width less than 7 cm required TAR, as opposed to 51.43% of patients in the laparoscopic subgroup. For widths between 7 and 10 cm, TAR rates were 43.48% in the robotic subgroup, as opposed to 100% in the laparoscopic group. According to a meta‐analysis, TAR was used alongside eTEP‐RS when there was a larger/more challenging defect or when there was difficulty in the median approximation of the margins of the posterior fascia [22]. Further, studies have also shown that robotic eTEP significantly reduces the need for extra component separations (13.7 vs. 67.4%; *P* < 0.001) [23]. We believe our finding of lower requirement of component separation is reflective of the fact that robotic surgery helps save more peritoneum and sac, enabling closure of the PRS–peritoneum complex without TAR in larger defects. In our practice, we close the PRS–peritoneum complex as a whole to provide a posterior cover to the mesh. Multiple studies have been published with this technique as opposed to PRS–PRS closure [24, 25, 26]. Wherever feasible, effort is made to preserve and recruit peritoneum from the sac and make it part of the posterior component, thus reducing TAR rates [27].

Additionally, EndoWrist instruments and ergonomics of the robotic platform allow surgeons to suture large anterior defects with just three ports, without component separation.

Because of the lower requirement of component separation, the total operating time was significantly lower in the robotic group for hernias < 7 cm and 7–10 cm. As an extension of the above argument, we report lower pain scores at 24 h (for defects < 7 cm) and 14 days postsurgery (for defects < 7 cm and 7–10 cm) in the robotic arm. In addition, within 14 days of surgery, the robotic group had higher patient well‐being scores for hernias < 7 cm.

We intend to report our long‐term results on recurrence and other outcomes in a separate publication.

## 5 Conclusion

In conclusion, the short‐term findings of this study demonstrate that the robotic‐assisted eTEP technique may be utilized to repair significantly larger hernia defects without component separation using TAR. However, short‐term follow‐up and retrospective design limit the interpretation of these results. Future multicentric studies with a prospective design and data on long‐term outcomes, including recurrence, will enable a thorough evaluation and future application of this technique.

## 6 Strengths and Limitations

**Strengths:** To the best of our knowledge, this is the first comparative study of robotic‐assisted and laparoscopic VHR in India. This is also one of a few global retrospective studies that compare robotic‐assisted eTEP versus laparoscopic eTEP for VHR. Only one experienced surgeon performed all surgeries, resulting in uniformity in the operative techniques.

**Limitations**: The retrospective design, lack of randomization, and absence of long‐term follow‐up precluded reporting medium‐ to long‐term outcomes. Additionally, the sample size needs to be bigger, and a multicenter study is needed to more accurately appreciate the differences between the two subgroups.

## Author Contributions

All the authors contributed to the collection of data. All the authors critically reviewed all manuscript drafts and provided comments. All the authors gave their approval for the final version to be published. The corresponding author is the guarantor of this study and, as such, takes full responsibility for the integrity of the data and the accuracy of the data analysis.

## Ethics Statement

All procedures performed in studies were in accordance with the ethical standards of the institutional ethics committee, the principles of the Declaration of Helsinki, and the applicable guidelines for good clinical practice (GCP). Ethical approval for this study was granted by the institutional ethics committee of Max Super Speciality Hospital, Vaishali (Ref. No. BHR/RS/MSSH/VSH/CRL/IEC/MAMBS/24‐06 dated 2^nd^ April 2024).

## Consent

Being a retrospective data collection study, the study data were collected in an anonymized fashion. Hence, informed consent was not applicable as per the national ethical guidelines.

## Conflicts of Interest

The authors declare no conflicts of interest.

## Data Availability

Data sharing does not apply to this article as no new data were created or analyzed in this study.
